# Chief Complaint: “There Is Something Burning in my Mouth”

**DOI:** 10.5811/cpcem.2018.8.39505

**Published:** 2018-09-28

**Authors:** Daniel Quesada, Silva Boyajian, Jagdipak Heer, Timothy Lin, Phillip Aguìñiga-Navarrete, Laura C. Castro

**Affiliations:** * Kern Medical, Department of Emergency Medicine, Bakersfield, California; † LAC+USC Medical Center, Department of Emergency Medicine, Los Angeles, California; ‡ Huntington Memorial Hospital, Department of Emergency Medicine, Pasadena, California

## CASE PRESENTATION

**Image 1:**
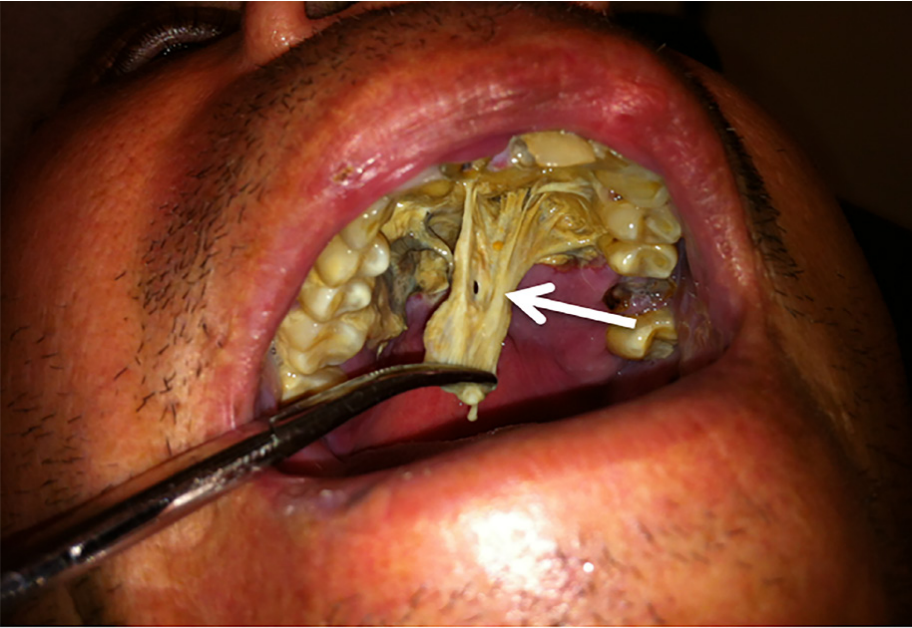
Demonstration of yellow, rubbery lesion found on the hard palate (white arrow) of the patient that upon biopsy revealed non-septated hyphae resembling Rhizopus species.

**Image 2:**
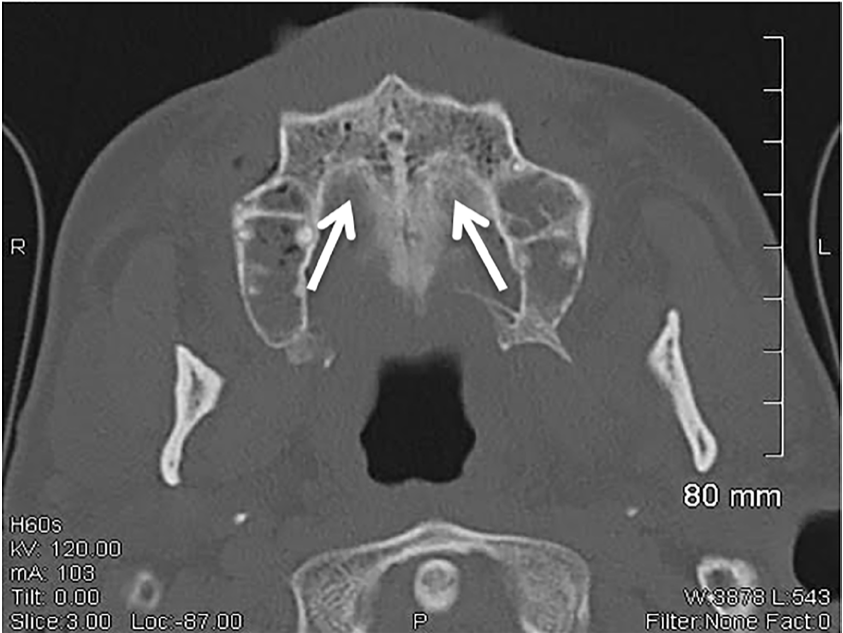
Axial view of a computed tomography scan of facial bones showing cortical irregularity of the hard palate, including submucosal emphysema (white arrows).

**Image 3:**
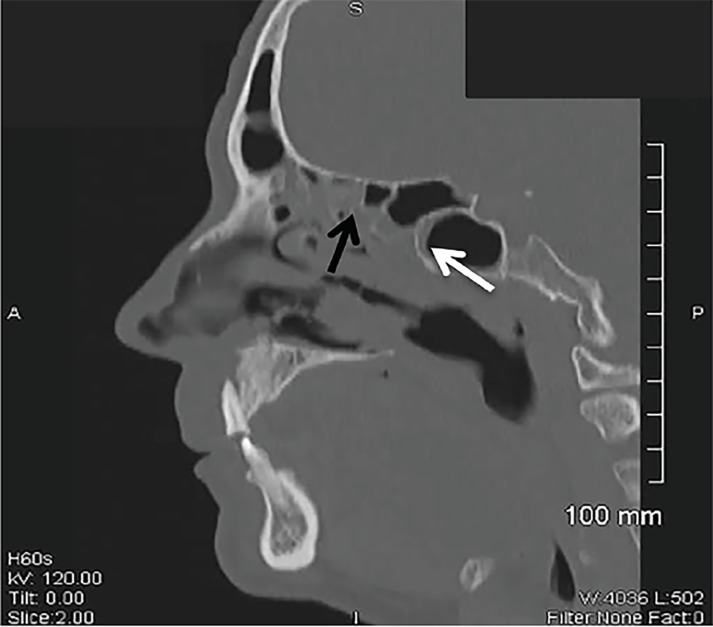
Sagittal view of a computed tomography scan of the facial bones revealed extensive acute and chronic sinusitis of the sphenoid (white arrow) and ethmoid sinuses (black arrow).

A 50-year-old Hispanic male with a history of diabetes presented to the emergency department with a painful maxillary mass for 12 days. He had been previously treated with antibiotics without improvement. Review of systems was significant for fever, diaphoresis, weight loss, and malodorous breath. Physical exam revealed poor dentition, mild tenderness to palpation of the maxillary sinuses and a 2.5 × 4 cm yellow, rubbery lesion on the hard palate (Image 1). The mass was pliable and adherent. Computed tomography of the face revealed irregularities of the hard palate, subcutaneous emphysema, and chronic sinusitis (Images 2 and 3).

## DIAGNOSIS

Rhinocerebral mucormycosis, an infection of the nasal and paranasal sinuses, is the most common presentation of the mucormycosis spectrum.^1^ Five hundred cases are reported in the United States each year.^2^ The fungi are found in dead and decaying matter such as soil but thrive in acidic glucose-rich environments.^1^,^3^ Infection begins with fungal seeding of the sinuses in an immunocompromised host (e.g., patients with malignancy, chronic steroid use, acquired immunodeficiency syndrome, and diabetes), who are predisposed due to decreased phagocytic activity of neutrophils and monocytes.^1^,^3^ From the sinuses, the fungus spreads to the orbits, oropharynx and mouth.^1^ When left untreated, *Mucor* can extend into the brain, cranial nerves, lungs, gastrointestinal system and kidneys, leading to vaso-occlusive thromboemboli, tissue infarction, and necrosis.^1^ Patients often present with indistinct symptoms such as headaches, low-grade fever, weakness, purulent nasal drainage, nasal congestion, nose bleeds, sinusitis, oral ulcers, and facial and periorbital pain.^1^

Our patient promptly received intravenous antifungals, including amphotericin B upon admission. Flexible laryngoscopy showed necrotic changes. A bilateral inferior maxillectomy was performed and a prosthetic palatal obturator was fitted for the patient. He remained on intravenous amphotericin B and later switched to oral posiconazole for completion of the six-month treatment.

**CPC-EM Capsule**

What do we already know about this clinical entity?

Rhinocerebral mucormycosis is the most common presentation of the mucormycosis spectrum and is most commonly found in immunocompromised individuals.

What is the major impact of the image(s)?

The images displayed are a visual demonstration of Mucor’s invasive abilities as well as the extent of bone destruction that it can cause.

How might this improve emergency medicine practice?

This case presentation reflects the significance of keeping a broad differential diagnosis, as a missed opportunity to diagnose this rare illness can result in death.

Documented patient informed consent and/or Institutional Review Board approval has been obtained and filed for publication of this case report.
